# Changes in the Saliva Proteome of Pigs with Diarrhoea Caused by *Escherichia coli*

**DOI:** 10.3390/proteomes11020014

**Published:** 2023-04-03

**Authors:** Miguel Rodrigues, Maria José López-Martinez, Alba Ortin-Bustillo, Jose Joaquin Cerón, Silvia Martinez-Subiela, Alberto Muñoz-Prieto, Elsa Lamy

**Affiliations:** 1 Department of Veterinary Medicine, School of Science and Technology, University of Evora, 7006-554 Evora, Portugal; 2 Interdisciplinary Laboratory of Clinical Analysis of the University of Murcia (INTERLAB-UMU), Department of Animal Medicine and Surgery, Veterinary School, Regional Campus of International Excellence Mare Nostrum, University of Murcia, 30100 Murcia, Spain; 3 Mediterranean Institute for Agriculture Environment and Development (MED), University of Evora, 7006-554 Evora, Portugal; 4 CHANGE-Global Change and Sustainability Institute, University of Evora, 7006-554 Evora, Portugal

**Keywords:** *E. coli*, salivary proteome, pigs, diarrhoea, lipocalin, ADA, biomarkers

## Abstract

*Escherichia coli* represents the main cause of diarrhoea in pigs. Saliva can provide information about the pathophysiology of diseases and be a source of biomarkers. We aimed to identify changes in the salivary proteome of pigs with diarrhoea caused by *E. coli*. Saliva samples were collected from 10 pigs with this disease and 10 matched healthy controls. SDS-PAGE (1DE) and two-dimensional gel electrophoresis (2DE) were performed, and significantly different protein bands and spots were identified by mass spectrometry. For validation, adenosine deaminase (ADA) was measured in 28 healthy and 28 diseased pigs. In 1DE, increases in lipocalin and IgA bands were observed for diseased pigs, whereas bands containing proteins such as odorant-binding protein and/or prolactin-inducible protein presented decreased concentrations. Two-dimensional gel electrophoresis (2DE) results showed that saliva from *E. coli* animals presented higher expression levels of lipocalin, ADA, IgA and albumin peptides, being ADA activity increased in the diseased pigs in the validation study. Spots containing alpha-amylase, carbonic anhydrase VI, and whole albumin were decreased in diseased animals. Overall, pigs with diarrhoea caused by *E. coli* have changes in proteins in their saliva related to various pathophysiological mechanisms such as inflammation and immune function and could potentially be biomarkers of this disease.

## 1. Introduction

Nowadays, saliva is considered an innovative and important source of biomarkers for many diseases in animals and humans. Overall, its composition can change due to stress, inflammation and alterations in the immune system or redox status, which can lead to the use of saliva analytes as biomarkers of pathological conditions [1]. This type of biological sample collection has many advantages, as it is painless and can be obtained by easy and non-invasive methods. In fact, saliva can be sampled without the need for specialized personnel in the field, anytime and anywhere [2]. Saliva is especially valuable in pigs, as in this species the collection of blood is stressful and painful for the animals [2].

It has been observed that saliva can show proteomic changes in sepsis experimentally induced by lipopolysaccharide (LPS) administration in pigs [3]. Aldolase A and serpin 12 were proteins in saliva that were significantly upregulated in sepsis. In addition, the proteome of saliva in pigs with *Streptococcus suis* infection has been studied, with the proteins metavinculin (VCL) and desmocollin-2 (DSC2) showing the highest relative abundance [4]. Moreover, proteomic changes have been reported in the saliva of pigs in situations of compromised welfare, with the proteins cornulin, heat shock protein 27, and lactate dehydrogenase (LDH) showing significant increases, and the immunoglobulin J chain showed a significant decrease [5].

*Enterotoxigenic Escherichia coli* (ETEC) is considered one of the main causes of diarrhoea in piglets [6], having a major economic impact on swine production [7]. ETEC produces several virulence factors, such as colonization factors (adhesins) and/or toxins. Colonization factors promote adherence to the host small intestine, and enterotoxins stimulate the lining of the intestine and induce watery diarrhoea [6], leading to sepsis [8]. Proteomic studies have been made to evaluate the changes in the intestine of pigs with *E. coli* diarrhoea [6,7] but, to our knowledge, no studies have been made in saliva.

The main objective of this study was to evaluate the possible changes in the salivary proteome of pigs with diarrhoea caused by *E. coli*, compared to healthy controls. To this end, SDS-PAGE and 2DE gel electrophoresis were used for the separation of proteins. After profile comparison, the mass spectrometry technique was used for the identification of the proteins differentially expressed between diseased and healthy animals. In addition, one protein showing significant changes in the proteomic study was selected for validation.

## 2. Materials and Methods

### 2.1. Population of Animals

For the proteomic studies, two groups of Large White weaning pigs from 6 to 9 weeks old were selected from commercial farms located in Southern Spain. One was a group of pigs diagnosed with diarrhoea caused by *E. coli* (*n* = 10, half males and half females), and the other were clinically healthy pigs (*n* = 10, half males and half females). The diseased animals had clinical signs compatible with this disease (diarrheic syndrome) and were positive for the presence of *E. coli* in rectal swabs following standard analytical procedures [9], being positive for *E. coli* F4 and heat-labile toxin. Additionally, 28 healthy pigs and 28 pigs with diarrhoea caused by *E. coli* from 6 to 9 weeks old were used for the validation study.

### 2.2. Saliva Collection and Sample Processing

A sponge was used for saliva collection. The pigs were allowed to chew on the sponge until it was thoroughly moist. Then, the sponges were placed in Salivette tubes (Sarstedt, Aktiengesellschaft & Co., D-51588 Nümbrecht, Germany) and kept at 4−8 °C until arrival at the laboratory, where the Salivette tubes were centrifuged at 3000× *g* and 4 °C for 10 min to obtain saliva supernatant. Saliva was transferred into the Eppendorf tubes and stored at −80 °C.

### 2.3. SDS PAGE

This technique was made according to a previously published procedure [10]. Proteins from individual saliva samples from all young animals (both healthy and diseased) were separated by SDS-PAGE gel electrophoresis on 12% acrylamide gels using Bio-Rad equipment (mini-protean, Bio-Rad, Alges, Portugal). Samples were carried out in duplicate to minimize technical errors. The total protein concentration of the samples was determined using the BCA assay (Thermo Scientific, Rockford, IL, USA). Briefly, a total of 9 µg of protein from each saliva sample was lyophilised and reconstituted with 40 µL of sample buffer (62.5 mM Tris-HCl pH 6.8, 2% (*w*/*v*) SDS, 10% glycerol, 5% DTT and bromophenol blue). Then, the samples were placed on ice and heated for 5 min at 98 °C to denature proteins. The Bio-Rad electrophoresis tank system was set up with running buffer (0.025 M Tris HCl, 0.192 M Glycine, and 0.1% (*w*/*v*) SDS; pH 8.3. Twenty µL of the reconstituted sample were applied to each lane (in duplicate), and electrophoresis was run at a constant voltage of 150 V until the dye front reached the end of the gel. The gels were fixed in 40% methanol, and 10% acetic acid for one hour, stained with Coomassie Brilliant Blue R-250 (0.2% in 40% methanol, 10% acetic acid) for another hour, and destained with 10% acetic acid several times until staining background remotion. Finally, LabScan software was used to acquire scanned images of the gels, and ImageLab software (Bio-Rad, Alges, Portugal) was used for gel analysis.

### 2.4. Two-Dimensional (2-DE) Gel Electrophoresis

For the 2DE technique, 3 pools of pig saliva samples were prepared from the group of healthy pigs and other 3 pools from the group of pigs with diarrhoea caused by *E. coli*. The volume of each individual corresponded to the same amount of total protein, in order to have a final total volume corresponding to 275 µg of total protein (determined using the BCA assay (Thermo Scientific, Rockford, IL, USA). Each pool was lyophilized and stored at −28 °C. The solid material was reconstituted with 250 µL of solubilization buffer [7 M urea, 2 M thiourea, 4% (*w*/*v*) 3-(3-cholamidopropyl) dimethylammonium propane sulfonate (CHAPS), 2% (*v*/*v*) ampholyte mixture (IPG buffer pH 3-11, GE Healthcare, Chicago, IL, USA), and 40 mM dithiothreitol (DTT)]. The mixture was incubated for 1 h at room temperature and subsequently centrifuged for 10 min at 10,000 rpm at room temperature. After this, the supernatant from each sample was divided into two volumes of 125 µL and applied in two different slots of the strip holder of the Multiphor II system (GE Healthcare, Chicago, IL, USA) to run each sample in duplicate. The last step in strip rehydration was to place the commercial gel strips [7 cm pH gradient 3–11 NL (IPG strips, GE Healthcare, Chicago, IL, USA)] in contact with the sample and leave them in passive rehydration overnight at room temperature, covered with mineral oil. Focusing was performed in a Multiphor II (GE, Healthcare, Chicago, IL, USA) at 12 °C with the following program (gradient): (1) 0–150 V for 15 min; (2) 150–300 V for 15 min; 300 V for 0.5 h; 300–3500 V for 4 h; 3500 for 3.5 h. Focused strips were equilibrated and applied on top of a sodium dodecyl sulphate-polyacrylamide gel electrophoresis (SDS-PAGE) on a 12% acrylamide gel and run at 150 V constant voltage on a mini-protein system (Bio-Rad, Alges, Portugal). Staining was made with CBB-R250. The image acquisition of the gels was made by a gel scanner (ImageScanner III, GE Healthcare, Chicago, IL, USA) and Lab scan software (GE Healthcare, Chicago, IL, USA), and the analysis was performed using the SameSpots software (v5.1.012, TotalLab, Gosforth, UK).

### 2.5. In-Gel Trypsin Digestion

After image analysis, the bands and spots that were observed to differ, in relative amounts, between healthy and *E. coli* individuals in SDS-PAGE and 2DE gels were selected for identification by MS. They were spliced into approximately 2 × 2 mm parts and distained. Then, they were alkylated and incubated with trypsin (Promega Corporation, Madison, MI, USA) and ProteaseMax surfactant (Promega Corporation, Madison, MI, USA) for 10 min at 4 °C. Finally, samples were digested at 37 °C for 16 h.

### 2.6. Protein Identification through HPLC-MS/MS Analysis

An HPLC/MS system consisting of an Agilent 1290 Infinity II Series HPLC (Agilent Technologies, Santa Clara, CA, USA) connected to an Agilent 6550 Q-TOF mass spectrometer (Agilent Technologies, Santa Clara, CA, USA) was used in this study. Parameters for the equipment analysis were set in MassHunter Workstation Data Acquisition software (Agilent Technologies, Rev. B.08.00, Santa Clara, CA, USA).

Dry samples from trypsin digestion were resuspended in a buffer with water/acetonitrile/formic acid and injected onto an Agilent AdvanceBio Peptide Mapping HPLC column, thermostated at 50 °C, at a flow rate of 0.4 mL/min.

The data processing and protein identification was made on Spectrum Mill MS Proteomics Workbench (Rev B.06.00.201, Agilent Technologies, Santa Clara, CA, USA). The criteria used for MS/MS search against the appropriate and updated protein database were: variable modifications search mode (carbamidomethylated cysteines, STY phosphorylation, oxidized methionine, and N-terminal glutamine conversion to pyroglutamic acid); tryptic digestion with 5 maximum missed cleavages; ESI-Q-TOF instrument (Agilent Technologies, Santa Clara, CA, USA); minimum matched peak intensity 50%; maximum ambiguous precursor charge +5; monoisotopic masses; peptide precursor mass tolerance 20 ppm; product ion mass tolerance 50 ppm; and calculation of reversed database scores.

### 2.7. Statistical Analysis

The data were evaluated for normal distribution using the Shapiro–Wilk test. Variables (protein concentration, protein bands and spots) for which normal distribution was not observed were transformed (log transformation). When normal distribution was achieved, Student’s *t*-test was used for group comparison, whereas non-normally distributed variables were compared using a non-parametric test (Mann–Whitney). Statistical analysis was performed with SPSS (v.28.0, IBM SPSS Statistics, New York, NY, USA). Statistically significant differences were considered when the *p*-value < 0.05.

### 2.8. Validation

Among the proteins identified with the relative abundance in saliva showing significant changes between healthy and diseased pigs, ADA was selected as a biomarker candidate for validation in an additional group of pigs with *E. coli* diarrhoea (*n* = 28), which was compared with a group of healthy pigs (*n* = 28). In both groups, half of the animals were male and half female.

The activity of ADA was measured using an automated assay that was previously validated in the saliva of pigs [11].

## 3. Results

### 3.1. Total Protein Concentration

The total protein concentration of saliva samples was observed to be significantly higher in *E. coli*-diseased animals compared to the healthy ones. Mean *E. coli* animals have almost 3 times higher values of total protein than healthy animals (76.4 ± 41.8 μg/mL vs. 280.5 ± 107.7 μg/mL, for healthy and *E. coli* groups, respectively; *p* = 0.001).

### 3.2. SDS-PAGE Profile

**Figure 1:**
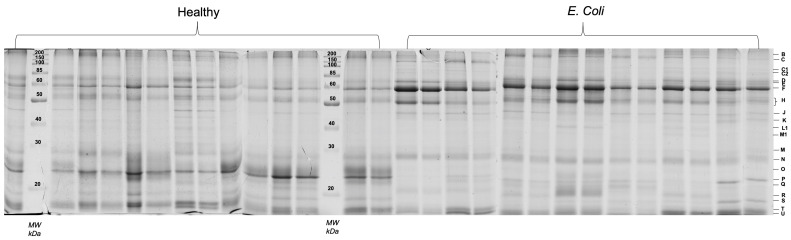
Salivary protein profiles (SDS-PAGE) of all the samples (healthy controls and *E. coli* diseased pigs). Each capital letter, on the right side, represents the bands compared between groups.

Salivary SDS-PAGE protein profiles allowed the constant visualization of clearly distinct 21 protein bands, with molecular masses between 10 and 200 kDa, whose levels were compared between groups (Figure 1).

Eight protein bands were observed to be differently expressed between healthy and diseased animals. Band C1 was a faint band, not identified through mass spectrometry, which was only observed in the *E. coli* group. The other 7 bands, although observed in animals from both groups, presented statistically significant differences, with bands B, H, M, N, and R increasing in diseased animals and bands P and T decreasing in those. The differences between groups, as well as mass spectrometry identifications of the proteins present in those bands, are presented in Table 1.

From the 1DE analysis, it was evident an increase in salivary lipocalin and IgA bands in *E. coli* diseased pigs, whereas bands containing proteins such as odorant-binding protein, a protease inhibitor from the submandibular origin and/or prolactin inducible protein were present in decreased levels in these animals.

### 3.3. Two-Dimensional Protein Profile (2-DE)

After gel analysis, it was possible to consider 127 protein spots constantly present in the different pool samples, which were compared between healthy and *E. coli* sample pools. Testing the possibility of separation of the two groups using principal component analysis, it is possible to see that the two components explain 46.98% of data variability (Supplementary Figure S1).

**Figure 2:**
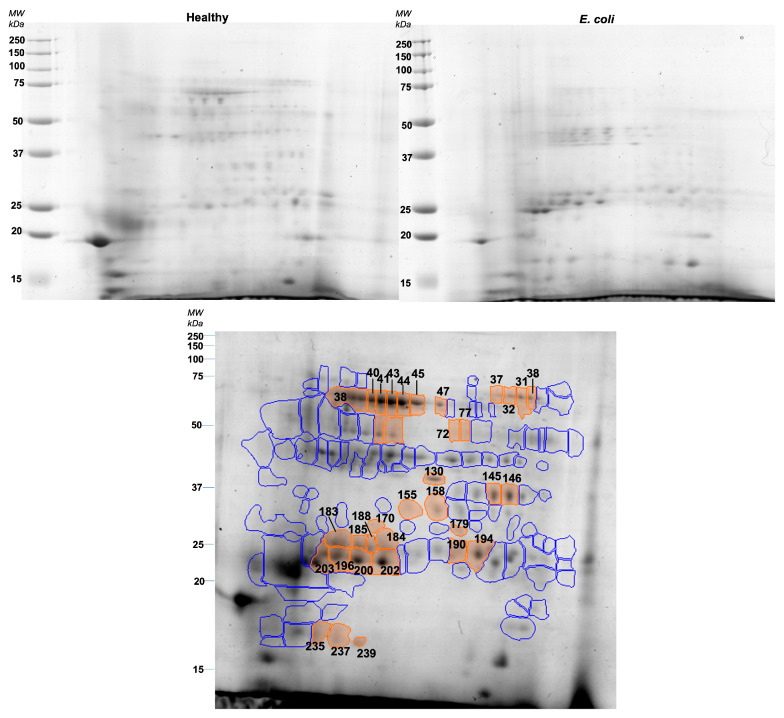
Representative gels of healthy (upper left) and *E. coli* (upper right) pools. The lower image represents the reference gel with protein spots differently expressed between groups (orange) and spots that did not show differences between groups (blue).

Through the between-subjects test (independent *t*-test), a total of 35 protein spots were observed to present a statistically significant difference (*p* < 0.05) (Figure 2). Among these, 15 protein spots were increased in *E. coli* animals, whereas 20 were decreased. The level of variation, as well as the salivary proteins identified, are presented in Table 2.

Taking together the 2DE results, it is possible to observe that *E. coli* pools presented higher expression levels of spots identified as lipocalin, adenosine deaminase, IgA, and albumin peptides. On the other hand, spots containing alpha-amylase, carbonic anhydrase, carbonate dehydratase VI, and whole albumin were decreased in pools from the diseased animals.

### 3.4. Validation

**Figure 3:**
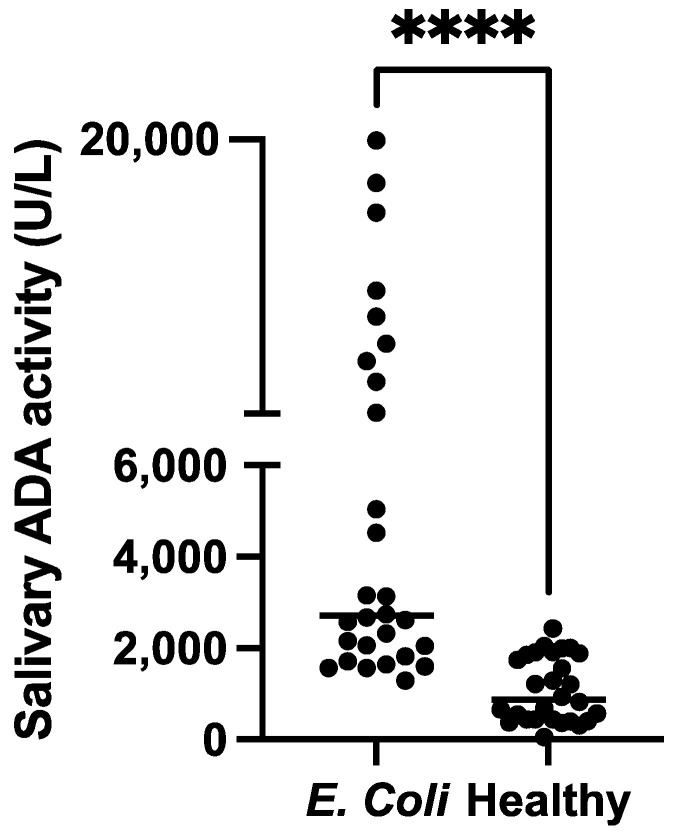
Comparison of the salivary adenosine deaminase activity (ADA) in pigs with diarrhoea caused by *E. coli* and healthy pigs. The plot shows the individual values of each group. **** *p* < 0.001.

The measurements of salivary ADA activity showed significantly higher activity levels in pigs with diarrhoea caused by *E. coli* (median 2712 U/L, minimum–maximum range 1293–19936 U/L) compared with healthy pigs (median 881.6 U/L, minimum–maximum range 60.8–2435 U/L) (*p* < 0.001) (Figure 3).

## 4. Discussion

In this report, changes in various proteins in the saliva of pigs with diarrhoea caused by *E. coli* were detected. To the authors’ knowledge, this is the first report in which a proteomic analysis of saliva is performed in pigs with diarrhoea due to *E. coli* infection and where changes in salivary proteins in this disease are described. The proteomic approach of this study used 1DE and 2DE gels. 1DE allows the separation of proteins only according to their molecular masses and the entry into the gel of a broad range of proteins, whereas 2DE may not be able to separate proteins with extreme isoelectric points or higher hydrophobicity. The lower requirement for total protein allowed testing samples at the individual level with this technique. On the other hand, 2DE allows for a more detailed protein profile, obtained after proteins are separated both by their charge and mass. Both 1DE individual samples and 2DE sample pools were run in duplicate to minimize the effect of technical errors inherent to the techniques.

From the 1DE analysis, it was evident that there was an increase in salivary lipocalin and IgA bands in *E. coli*-diseased pigs, whereas bands containing proteins such as odorant-binding protein and/or prolactin-inducible protein were present in decreased concentrations in these animals.

Lipocalin (LCN) family proteins are small proteins (18–40 kDa) expressed in numerous tissues and involved in multiple processes (i.e., inflammation, detoxification, and immune activation) by transporting hydrophobic molecules (e.g., steroids, retinoids, or lipids) to cells [12]. Some members of this family of proteins such as lipocalin-2 (also known as neutrophil gelatinase-associated lipocalin) are considered acute phase proteins showing increases in inflammation [13]. Lipocalin-2 is increased in the serum of humans with inflammatory bowel disease and is correlated with the activity of this disease [14,15]. In addition, it has been described to capture bacterial siderophores produced by pathogenic bacteria, such as *E. coli* and, indeed, Lcn2-deficient mice are prone to infection and sepsis [16]. Although in our study LCN increased, in a previous report it was observed a decrease of LCN in the saliva of pigs with *Streptococcus suis* infection [4]. Further studies should be undertaken to elucidate the mechanisms involved in the change in LCN since in some cases, such as in the *Streptococcus suis* infection, the decrease of lipocalin could indicate a high susceptibility to worsening sepsis [4].

Odorant binding protein (OBP) is involved in olfaction and defence against oxidative injury. In addition, this protein has been related to inflammation, showing a decrease in lungs in bovine after LPS administration. This decrease in OBP levels may be an additional mechanism to allow inflammatory mediators to stimulate neutrophil recruitment and oxidative burst in the lung and possibly in other tissues [17].

Prolactin-inducible protein (PIP) is a small (17 kDa) single polypeptide chain protein expressed in various human body parts, including the salivary gland, lacrimal gland, trachea, prostate, muscle, mammary glands, and lungs [18]. Its expression is upregulated by prolactin and androgens, and oestrogens downregulate it. It is involved in the immune response and can inhibit the growth of bacterial species [19]. The decrease in PIP found in our study could be related to a decrease in prolactin, which has been described in pigs with inflammation [20] and humans with sepsis [21].

In 2DE, lipocalin, adenosine deaminase (ADA), IgA, and albumin peptides were increased in the saliva of pigs with *E. coli*, whereas spots containing carbonic anhydrase, carbonic dehydratase VI, alpha-amylase, and whole albumin were decreased in pools from the diseased animals. ADA was selected to validate the proteomic results due to the existence of an automated assay validated for pigs [22]. ADA increases inflammation and sepsis in the saliva of pigs [4,11]. The increase in ADA found in our proteomic study was also confirmed in the larger population of pigs with diarrhoea with *E. coli* compared to healthy pigs, corroborating the higher levels of this protein in saliva in this disease, possibly reflecting activation of inflammation and the immune system. In addition, IgA, which is produced by the immune system to prevent the invasion of pathogenic microbes and is found in large amounts in the mucosal secretions of the gastrointestinal tract and saliva, was increased in our study. This could agree with other reports that have described an increase in IgA in mucosal secretions after an *E. coli* infection [23].

Carbonic anhydrase (CA; EC 4.2.1.1) represents a group of enzymes that catalyse the reversible hydration/dehydration of CO_2_ and water. It is involved in the regulation of colonic electrolyte transport and inhibition of CA activity in the colonic mucosa can lead to a decrease in water absorption [14,15]. In addition, CA has been suggested to mediate the colonic absorptive response to changes in systemic acid-base balance. In this line, human patients with mild or moderate ulcerative colitis showed a significant reduction of the CA isoenzyme I mRNA and protein and total CA activity in the inflamed mucosa compared to controls [24]. Therefore, it could be postulated that the decreases in CA found in our report would be related to damage in the intestinal mucosa. Carbonic dehydratase VI, which is considered an isoenzyme of CA, was also decreased in our study, possibly due to the reasons described above.

A decrease in spots containing alpha-amylase was also observed in the diseased animals. Usually, the activity of alpha-amylase in the saliva is increased in situations of stress and disease in pigs [25]. The divergence of the decrease found in the amount of amylase in our study compared with the increases in the activity reported in other diseases could be due to the divergences between the amount of one enzyme and its activity, which can occur especially in the case of alpha-amylase [26]. In fact, the 2DE spots represent the relative amount of the forms of the protein, which may not be the ones most contributing to the enzymatic activity. Regarding the albumin, there was a decrease in whole albumin but an increase in peptides with MW lower than the MW of the primary form of albumin. This could indicate that albumin could have some proteolysis in the saliva of diseased pigs. Increases in albumin fragments in the blood due to albumin proteolysis have been described in some diseases such as renal failure [27].

Overall, in our report, we found changes in proteins in saliva related to inflammation and the immune system, as have been described in saliva in pigs with sepsis experimentally induced by LPS administration and other infectious diseases such as *S. suis* infection [3,4].

This report has a limitation in the use of pools for 2D, which does not accurately represent the contribution of the different individual samples. However, there was an agreement in proteins such as lipocalin and IgA between the results of 1D (that was made in individual samples) and 2D gels; also, the increases in ADA in 2D gels were later confirmed by an automated assay in a larger number of individual samples. Further studies involving the validation of a larger number of proteins and a larger number of animals should be made to corroborate the results of our report. In this line, although the study of diseased animals on farms provides a real picture of the disease under field conditions, ideally additional studies in which *E. coli* infection is induced in experimental pigs should be performed to confirm the findings of this report. In addition, it would be of interest to perform additional studies to evaluate possible different proteoforms and protein species to better elucidate the proteome complexity in the saliva of healthy pigs and pigs with diarrhoea caused by *E. coli*.

## 5. Conclusions

It can be concluded that pigs with diarrhoea caused by *E. coli* infection have changes in proteins in their saliva that can be detected by gel proteomics. These proteins are related to various pathophysiological mechanisms activated in diseases such as inflammation and immune function, and could potentially be biomarkers that could help detect and monitor this disease.

## Figures and Tables

**Table 1:** Differences in protein band expression levels (mean ± standard deviation of %Vol) between *E. coli* diseased and healthy pigs and correspondent protein identification and MS.

Band	Healthy	*E. coli*	*p*-Value	UNIPROT Protein Accession Number	Protein (Entry Name)	Seq Coverage (%)	ID Score	Theoretical MW (kDa)	Apparent MW (kDa)
B	1.62 ± 0.80	5.36 ± 3.06	0.001	018758	Submaxillary apomucin	1.3	238.4	1184.1	>200 kDa
C1	-				ni				120
H	3.74 ± 0.59	9.77 ± 2.91	0.0005	A0A287B626	IgA constant region	39.3	209.6	44.2	54
M ^#^	1.35 ± 1.09	2.94 ± 0.75	0.015	A0A0A0MY58 and F1SN92	Immunoglobulin heavy constant mu and Salivary lipocalin	28.5 and 25.1	75.1 and 43.5	32.7 and 21.6	28.5
N	6.88 ± 2.44	10.20 ± 1.43	0.009	F1SN92	Salivary lipocalin	54.9	152.5	21.6	26
P	17.51 ± 4.27	3.40 ± 2.10	0.0005	P81245	Odorant-binding protein	75.1	199.5	17.7	18
R	1.22 ± 1.63	4.00 ± 2.47	0.033	A0A4X1TU02	Salivary lipocalin	57.5	143.4	21.6	16.5
T ^#^	14.15 ± 4.91	8.33 ± 4.70	0.043	A0A286ZRW6 and A0A287ASS4	Double-headed protease inhibitor, submandibular gland-like and Prolactin inducible protein	29.4 and 36	58.31 and 56.35	13.3 and 12.4	13

ni—protein failing identification by MS; # in the tryptic mixture, peptides corresponding to more than one protein were observed in the spectra, indicating that more than one protein was present in the band.

**Table 2:** Protein spots differently expressed between healthy and *E. coli*-diseased pigs.

Spot Number	Fold Change	Group with Higher Level	*p*-Value	Protein (Entry Name)	UNIPROT Protein Accession Number	Seq Coverage (%)	ID Score	Theoretical MW (kDa)	Apparent MW (kDa)
237	4.24	*E. coli*	5.24 × 10^–5^	Adenosine deaminase and salivary lipocalin	A0A0B8RW47 and A0A4X1TU02	22.5 and 15.7	39.5 and 23.8	40.9 and 21.6	17.5
33	1.72	Healthy control	0.000222	n.i.					
185	2.30	*E. coli*	0.00063	IgA constant region	A0A287B626	3.8	23.6	44.2	27.5
188	2.41	*E. coli*	0.000733	IgA constant region	A0A287B626	2.6	20.0	44.2	27.5
41	3.29	Healthy control	0.000763	Albumin (whole)	A0A286ZT13	41.1	327.8	68.2	74.5
145	1.56	Healthy control	0.000794	Carbonate dehydratase VI	A0A4X1W7S7	15.1	39.5	34.7	36.0
40	2.72	Healthy control	0.000871	Albumin (whole)	A0A286ZT13	41.1	327.8	68.2	74.5
202	2.28	*E. coli*	0.000887	Ig-like domain-containing protein	A0A287A4Y3	15.4	41.4	24.7	26.0
44	3.03	Healthy control	0.001118	Lactoperoxidase	A0A480RK36	6.6	48.7	80.3	74.5
196	2.97	*E. coli*	0.001675	Albumin (fragment) and salivary lipocalin	A0A286ZT13 and A0A4X1TU02	13.6 and 23.5	100.5 and 31.5	68.2 and 21.6	26.0
200	2.34	*E. coli*	0.002233	Albumin (fragment) and salivary lipocalin	A0A286ZT13 and A0A4X1TU02	13.6 and 23.5	100.5 and 31.5	68.2 and 21.6	26.0
45	2.10	Healthy control	0.002679	Lactoperoxidase	A0A480RK36	6.7	48.8	80.3	74.5
43	3.30	Healthy control	0.003706	Lactoperoxidase and polymeric immunoglobulin receptor	A0A480RK36 and A0A0E3M2Q4	7.5 and 6.5	45.5 and 37.3	80.3 and 67.3	74.5
194	1.71	*E. coli*	0.004477	Albumin (fragment)	A0A286ZT13	7.8	64.3	68.2	26.5
31	2.29	Healthy control	0.005324	n.i.					
47	1.66	Healthy control	0.005441	Lactoperoxidase and polymeric immunoglobulin receptor and	A0A0E3M2Q4 and A0A480RK36	12.3 and 4.9	86.1 and 34.7	67.3 and 80.3	74.0
184	1.86	*E. coli*	0.007066	n.i.					
203	2.94	*E. coli*	0.007897	Ig-like domain-containing protein	A0A287A4Y3	18.5	34.0	24.7	26.0
38	2.12	Healthy control	0.009251	Albumin (whole)	A0A286ZT13	41.1	327.8	68.2	101.0
32	1.50	Healthy control	0.012577	n.i.					
37	2.03	Healthy control	0.01381	n.i.					
155	1.51	Healthy control	0.015799	Carbonic anhydrase	A0A4X1W9S1	11.0	27.7	36.3	36.0
179	1.96	Healthy control	0.020918	Carbonate dehydratase VI	A0A4X1W7S7	11.5	47.2	34.7	27.5
73	1.78	Healthy control	0.021757	Alpha-amylase	F1S573	30.1	146.0	55.8	58.0
74	1.39	Healthy control	0.026339	Alpha-amylase	F1S573	30.9	123.4	55.8	58.0
235	2.49	*E. coli*	0.030702	Adenosine deaminase and salivary lipocalin	A0A0B8RW47 and A0A4X1TU02	22.5 and 15.7	39.5 and 23.8	40.86 and 21.61	18.0
130	2.26	*E. coli*	0.033046	n.i.					
146	1.38	Healthy control	0.037883	Carbonate dehydratase VI	A0A4X1W7S7	9.8	25.6	34.7	36.0
77	1.67	Healthy control	0.039092	n.i.					
190	1.75	*E. coli*	0.040238	Albumin (fragment)	A0A286ZT13	9.2	61.0	68.2	26.0
239	2.94	*E. coli*	0.042094	Salivary lipocalin	F1SN92	4.4	24.51	21.6	17.5
170	1.36	*E. coli*	0.046073	n.i.					
72	2.01	Healthy control	0.046326	n.i.					

Note: n.i. means spots that were not identified.

## Supplementary Materials

The following supporting information can be downloaded at: https://www.mdpi.com/article/10.3390/proteomes11020014/s1, Figure S1: Distribution of sample pools among the two first components obtained by principal component analysis.
