# Supplementary figures and images for: Changes in the Saliva Proteome of Pigs with Diarrhoea Caused by Escherichia coli
Source: Proteomes. 2023 Apr 3;11(2):14. doi: 10.3390/proteomes11020014 (PMC10123737; doi:10.3390/proteomes11020014)

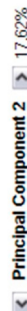

Principal Component 1 29,36%

Supplement: Supplementary file 1 [file proteomes-11-00014-s001.zip › proteomes-2275746-supplementary.pdf]
